# Aurora-A affects radiosenstivity in cervical squamous cell carcinoma and predicts poor prognosis

**DOI:** 10.18632/oncotarget.15663

**Published:** 2017-02-24

**Authors:** Yuhua Ma, Jie Yang, Ruozheng Wang, Zegao Zhang, Xiaoli Qi, Chunhua Liu, Miaomiao Ma

**Affiliations:** ^1^ Radiotherapy Second Department, People's Hospital of Xinjiang Uyghur Autonomous Region, Urumqi, Xinjiang, China; ^2^ The Department of Radiation Oncology, Tumor Hospital Affiliated To Xinjiang Medical University, Urumqi, Xinjiang, China

**Keywords:** Aurora kinase A, cervical squamous cell carcinoma, radiation, Uyghur, cell cycle

## Abstract

**Background:**

Definitive radiation therapy (RT) (with or without cisplatin-based chemotherapy) is one of the most effective treatments for cervical squamous cell carcinoma (CSCC), but efficacy is limited due to resistance. In the present study, we investigated the relationship between the expression of Aurora kinase A (Aurora-A, AURKA)and response to RT in patients with CSCC.

**Methods:**

The expression of Aurora-A in biopsy specimens of untreated primary tumors in 129 Uyghur patients with CSCC was investigated immunohistochemically. Primary treatment in these patients was definitive radical RT, which consisted of pelvic RT plus brachytherapy (total point A dose:70–85 Gy) (with or without cisplatin-based chemotherapy). The prognostic value of tumoral Aurora-A expression and patients’ clinical outcomes were evaluated.

**Results:**

Aurora-A expression was significantly associated with lymph node metastasis (P<0.001), large tumor size (P<0.001), low hemoglobin (Hb) level (P=0.011) and recurrence (P<0.001), but not other clinicopathological factors. Definitive RT was unfavorable in patients with high Aurora-A expression (P < 0.001). In 129 enrolled patients, lymph node metastasis, large tumor size, low Hb level, and AURKA overexpression were prognostic factors for both recurrent free survival (RFS) and overall survival (OS) in univariate analysis. However, only high AURKA expression was an adverse independent risk factor for both RFS (hazard ratio, 3.953; 95% CI, 1.473-10.638; P = 0.006) and OS (hazard ratio 9.091; 95%CI 2.597-32.258; P<0.001) in multivariate analyses.

**Conclusions:**

Aurora-A may serve as a predictive biomarker of radiation response and a therapeutic target to reverse radiation therapy resistance.

## INTRODUCTION

Cervical cancer (CC) is the fourth most common malignant tumor in women worldwide, with 528,000 new cases in 2012 [1]. Approximately 87% of CC cases occur in developing countries. Furthermore, the morbidity rate due to CC in China is among the highest in the world [2]. In particular, Uyghur women who live in the southern region of Xinjiang Province, China, have the highest morbidity (590/100,000) [3] due to CC in the country. Squamous cell carcinoma (SCC) accounts for approximately 95% of all CCs [4]. Furthermore, CC tends to develop in Uyghur women at a younger age.

Concurrent chemoradiation therapy (CCRT) with cisplatin is generally the primary treatment of choice for stage IB_2_ to IVA disease based on the results of five randomized clinical trials [5, 6]. These five trials showed that CCRT resulted in a 30% to 50% decrease in the risk of death compared with radiation therapy (RT) alone [7–11]. Although RT can achieve a good outcome in patients with early-stage disease, treatment failure occurs in patients with advanced-stage disease. Studies have shown that the failure rate in patients with stage I–IVA CSCC after definitive RT is 29% [12]. In addition, although chemoradiation is tolerated, acute and long-term side effects have been reported [13]. For locally advanced CC, several criteria have been proposed to predict the risk of recurrence. These include age [14], adenocarcinoma [14], stage [14], tumor size [15, 16], and pretreatment anemia [16]. However, even in patients with similar sized tumors, the same stage of CSCC and receiving the same dose of radiotherapy, treatment response can be different. Thus, there is an urgent need to identify new biomarkers and/or therapeutic targets that can be used to treat these patients.

Aurora-A, an important member of the Aurora kinase family, is mainly located in the central body at prophase, near the pole spindle at the medium-term, and located at pole microtubules at anaphase and telophase [17]. Aurora-A regulates the functions of centrosomes, spindles and kinetochores required for correct mitotic progression. Aurora-A has been observed to positively regulate the G2 to M phase of the cell cycle, and activation of Aurora-A in late G2 is inhibited by DNA damage [18]. The cell cycle significantly influences radiosensitivity. In the present study, we determined the expression of Aurora-A in Uyghur CSCC patients treated with definitive radical RT and determined its correlation with clinical characteristics. We also investigated the relationship between the expression of Aurora-A and the response to RT or CCRT in patients with CSCC. In addition, we identified the expression of Aurora-A and its correlation with prognosis in CSCC.

## RESULTS

### Aurora-A staining and its association with clinicopathological characteristics

**Figure 1:**
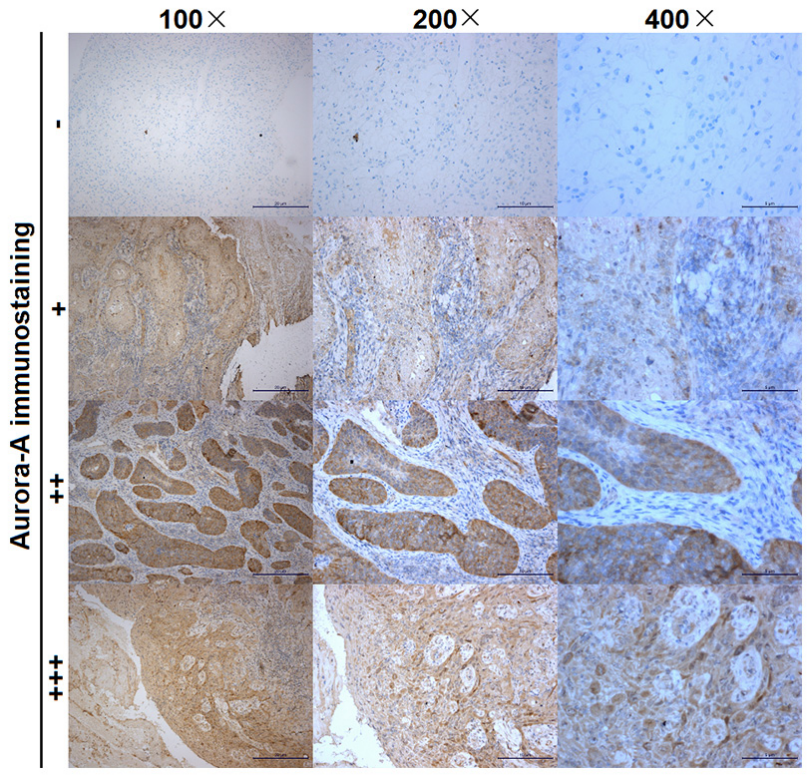
Expression of AURKA in CSCC The intensity of the dye color was graded as 0 (no color), 1 (light yellow), 2 (light brown) or 3 (brown), and the number of positive cells was graded as 0 (no staining), 1 (<30%), 2 (30%-60%) and 3 (>60%); the two grades were added together and the specimens were assigned to one of four levels as follows: a score of 0-1 (-), a score of 2 (+), a score of 3-4 (++), a score of more than 5 (+++). Immunostaining was considered to be low (-, +) or high (++, +++).

AURKA expression was analyzed by immuno-histochemical (IHC) staining on tissues. Aurora-A expression was mainly found in the cytoplasm of tumor cells (Figure 1), which was similar to a previous report [19]. High expression of Aurora-A was detected in 73 out of 129 (56.6%) selected CSCC tissues and low in the remaining 56 (43.4%) tissue specimens. Basic clinicopathological characteristics of the 129 patients are shown in Table 1. Fifty-eight patients (44.96%) experienced recurrence, including 14 locoregional relapses, 29 distant metastases, and 15 multiple site recurrences. Approximately 50% of patients had lymph node metastasis and more than half had large tumors (≥5.7 cm). Approximately 30% of these patients had pretreatment anemia. AURKA expression was significantly correlated with lymph node metastasis (P<0.001), large tumor size (P<0.001), low Hb level (P=0.038), and recurrence (P<0.001).

**Table 1:** Clinicopathological characteristics of 129 CSCC patients and their association with Aurora kinase A (AURKA) IHC intensity

Clinicopathological features	NO	AURKA IHC		χ2	*P*
		High(++,+++)	Low(-,+)		
ECOG performance status					
(0,1)	87	49	38	0.010	0.919
(2,3)	42	24	18		
Age (y)					
<50	55	29	26	0.340	0.560
≥50	74	44	30		
Stage					
II	61	37	24	0.497	0.481
III	68	36	32		
Lymph nodes metastases					
No	70	28	42	15.701	0.000
Yes	59	45	14		
Differentiation					
Well-moderate	62	39	23	1.474	0.225
Poor	67	34	33		
Tumor size					
≥5.7cm	83	64	19	39.894	0.000
<5.7cm	46	9	37		
SCC-ag level (ng/ml)					
<2	48	24	24	1.352	0.509
2-10	53	32	21		
<10	28	17	11		
Hb level (g/dl)					
<10	35	25	10	4.306	0.038
≥10	94	48	46		
Treatment					
Radiation	55	34	21	0.728	0.393
Concurrent Chemoradiation	74	39	35		
Recurrence events					
Yes	58	49	9	31.345	0.000
No	71	24	47		

### Relationships between Aurora-A expression and response to RT

The percentage of patients with a complete response (CR), partial response (PR)/stable disease (SD) or progressive disease (PD) following RT was: 61.24% (79 out of 129), 33.33% (43 out of 129) and 5.43% (7 out of 129), respectively. Analysis of the relationship between the expression of Aurora-A and clinical response to RT indicated that RT was more favorable in patients who had low-Aurora-A expression in tumors (P< 0.001) (Table 2).

**Table 2:** correlation of aurora-A expression with clinical response to RT

AURKA IHC	Clinical response to RT (n=129)			total	*χ2*	*P*
	CR	PR+SD	PD			
high	33	34	6	73	18.324	*P*<0.001
low	46	9	1	56		

### Relationship between Aurora-A expression and RFS or OS

The 5-year RFS and OS were 23.26% and 27.91%, respectively.

**Figure 2:**
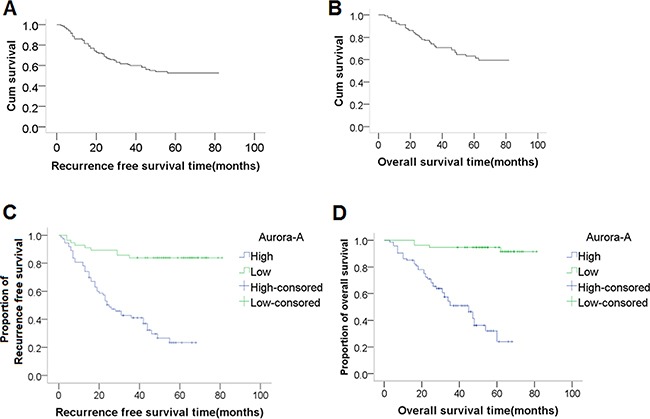
Recurrence-free survival (RFS) and overall survival (OS) curves, and the association between AURKA expression and survival RFS curve **(A)** and OS curve **(B)**, respectively, and the 5-year RFS and OS were 23.26% and 27.91%, respectively. **(C-D)** Kaplan-Meier plots of **(C)** RFS and **(D)** OS in 129 patients with CSCC according to AURKA expression. The *P* values for survival comparison, which were obtained by log-rank test, were all less than 0.05.

Univariate analysis showed that the RFS in all 129 cases was significantly influenced by lymph node metastasis (P<0.001), large tumor size (P<0.001), low Hb level (P=0.011), and AURKA expression level (P<0.001) (Table 3). The Kaplan-Meier RFS curves according to AURKA expression are shown in Figure 2. However, only AURKA overexpression (hazard ratio, 3.953; 95% CI, 1.473-10.638; P = 0.006) was identified as an independent unfavorable prognostic factor for RFS in multivariate analysis (Table 3). Univariate analysis showed that the OS of all 129 cases was significantly influenced by lymph node metastasis (P<0.001), large tumor size (P<0.001), low Hb level (P=0.004), and AURKA expression (P<0.001) (Table 4). The Kaplan-Meier OS curves according to AURKA expression level are shown in Figure 2. However, only AURKA overexpression (hazard ratio 9.091; 95%CI 2.597-32.258; P<0.001) was identified as an independent unfavorable prognostic factor for OS in multivariate analysis (Table 4).

**Table 3:** Univariate and multivariate analyses of the prognostic influence of clinicopathological factors on recurrence-free survival

Clinicopathological features	median RFS (month)	Univariate analyses					Multivariate analyses			
		RFS (%)			*χ*2	P	HR	95.0% CI for HR		P
		1year	3year	5year				low	high	
ECOG performance status										
(0,1)	72.00	81.61	58.62	19.54	0.637	0.627				
(2,3)	45.72	95.24	50.00	30.95						
Age (y)										
<50	72.00	83.63	63.64	27.27	3.715	0.054				
≥50	44.19	87.84	50.00	20.27						
Stage										
II	72.00	86.89	62.30	22.95	0.532	0.466				
III	72.00	85.29	50.00	23.53						
Lymph nodes metastases										
No	72.00	92.85	77.14	40.00	31.566	0.000	0.658	0.316	1.368	0.262
Yes	22.31	77.97	30.51	3.39						
Differentiation										
Well-moderate	72.00	90.32	50.00	20.97	0.002	0.961				
Poor	72.00	82.09	61.19	25.37						
Tumor size										
≥5.7cm	32.42	80.72	39.76	13.25	16.056	0.000	1.508	0.72	3.165	0.276
<5.7cm	72.00	95.65	84.78	41.30						
SCC-ag level (ng/ml)										
<2	54.41	89.58	52.08	25.00	0.365	0.833				
2-10	72.00	79.25	60.38	22.64						
>10	72.00	92.86	53.57	21.43						
Hb level (g/dl)										
<10	23.25	80.00	45.71	20.00	4.283	0.038	0.879	0.504	1.531	0.647
≥10	72.00	88.30	59.57	24.47						
Treatment										
Radiation	52.78	90.91	54.55	29.09	0.307	0.580				
Concurrent Chemoradiation	72.00	82.43	56.76	18.92						
Aurora-A IHC										
High	27.60	80.82	34.25	4.11	35.878	0.000	3.953	1.473	10.638	0.006
Low	72.00	92.86	83.93	48.21						

**Table 4:** Univariate and multivariate analyses of the prognostic influence of clinicopathological factors on overall survival

Clinicopathological features	median OS (month)	Univariate analyses					Multivariate analyses			
		OS (%)			*χ*2	P	HR	95.0% CI for HR		P
		1year	3year	5year				low	high	
ECOG performance status										
(0,1)	72.00	90.80	65.52	24.14	0.019	0.891				
(2,3)	72.00	95.24	64.28	35.71						
Age (y)										
<50	72.00	94.55	69.09	29.09	2.375	0.123				
≥50	70.89	90.54	62.16	27.03						
Stage										
II	72.00	96.72	68.85	26.23	3.210	0.073				
III	72.00	88.24	61.76	29.41						
Lymph nodes metastases										
No	72.00	100.00	85.71	48.57	31.257	0.000	0.869	0.411	1.835	0.712
Yes	32.77	84.75	40.68	3.39						
Differentiation										
Well-moderate	72.00	93.55	58.06	25.81	0.028	0.867				
Poor	72.00	91.04	71.64	29.85						
Tumor size										
≥5.7cm	53.24	87.95	48.19	16.87	23.076	0.000	2.646	0.987	7.092	0.053
<5.7cm	72.00	100.00	95.65	47.83						
SCC-ag level (ng/ml)										
<2	70.99	97.92	62.50	31.25	1.101	0.577				
2-10	72.00	84.91	66.04	24.53						
>10	72.00	96.43	67.86	28.57						
Hb level (g/dl)										
<10	72.00	91.49	71.28	29.79	6.466	0.004	0.704	0.389	1.276	0.247
≥10	48.95	94.29	48.57	22.86						
Treatment										
Radiation	72.00	90.54	62.16	22.97	0.009	0.925				
Concurrent Chemoradiation	72.00	94.55	69.09	34.55						
AURKA IHC										
High	38.44	86.30	42.47	5.48	44.402	0.000	9.091	2.597	32.258	0.001
Low	72.00	100.00	94.64	57.14						

## DISCUSSION

Aurora-A plays a key role in the regulation of cell cycle progression and those relating to cell cycle and mitosis control are associated with worse clinical outcomes in early-stage ovarian cancer [20]. Aurora-A protein is overexpressed in many tumors and this overexpression is associated with unfavorable prognosis and low survival [21–24]. At present, definitive radical radiotherapy is the main treatment for patients with locally advanced CC. Considering radiation response is one of the most important factors in predicting the prognosis of these patients, it is very important to identify predictive biomarkers in clinical practice. To date, the expression of Aurora-A and its prognostic significance in CC has been poorly investigated. In 2008, a study [25] showed that Aurora A expression was significantly increased in carcinoma and cervical intraepithelial neoplasia 3, compared with normal cervix, and this overexpression was a relatively early phenomenon in the genesis of malignant epithelial tumorigenesis. In 2009, another study [26] showed that the expression of Aurora-A mRNA and protein was significantly higher in cervical carcinoma cells than in normal cervical epithelial cells. Patients with high Aurora-A expression had poorer disease-free survival and OS rates than patients with low Aurora-A expression. However, there are no data on its potential role in predicting CC radiotherapy response.

In this study, we determined tumoral Aurora-A expression using 129 tissue specimens from CSCC patients treated with definitive radical RT. High Aurora-A expression was found in 56.57% (73/129) of patients, this percentage is very similar to that previously reported by Zhang et al. who showed that 51.3% (38/74) of CC tissues examined demonstrated mRNA expression by reverse transcription-polymerase chain reaction (RT-PCR) [26]. Our results showed that increased expression of Aurora-A was significantly associated with aggressive tumor variables, including lymph node metastasis, large tumor size, low Hb level and disease recurrence. These findings are consistent with those in previous reports, as one study found that Aurora-A was overexpressed in laryngeal squamous cell carcinoma and was associated with advanced tumor stage [27]. Another report [28] showed that Aurora-A mRNA and protein up-regulation was significantly associated with tumor stage and the occurrence of regional lymph node metastasis, as well as distant metastasis.

In multivariate analysis, high Aurora-A expression was an independent adverse risk factor for both RFS and OS in CSCC patients treated with definitive radical RT. In addition, our study suggested that patients with high Aurora-A expression may benefit less from RT treatment, as it was associated with poorer treatment response and shorter RFS and OS. Thus, we believe Aurora-A is a potential biomarker for predicting unfavorable radiation response and prognosis in CSCC patients.

Consistent with our findings, a recent randomized controlled trial, semiquantitatively evaluated Aurora-A expression in 144 cases with locally advanced naso-pharyngeal carcinoma (NPC) by immunohistochemistry staining. Of these patients, 69 received neoadjuvant chemotherapy plus CCRT, and 75 cases were treated with neoadjuvant chemotherapy plus RT. It was found that Aurora-A was highly expressed in NPC, but was deficient in normal adjacent epithelia; Aurora-A overexpression predicted a shorter 5-year OS, progression-free survival, and distant metastasis-free survival, and multivariate regression analysis confirmed that Aurora-A was an independent prognostic factor for death, recurrence, and distant metastasis; these results confirmed that Aurora-A was an independent prognostic factor for NPC patients who underwent RT [29]. Another report showed that Aurora-A overexpression was an independent prognostic factor for LSCC and was responsible for the relative tumor resistance to radiation therapy [27]. However, the results of another study [30] were in contrast to those of our study and showed that patients with Aurora-A overexpression had better clinical and histological response to CCRT. In some *in vitro* studies, inhibition of Aurora-A potently inhibited proliferation of atypical teratoid/rhabdoid tumor cells [31], glioblastoma neurosphere tumor cells [32], canine mast cell tumor cells [33] and sensitized these cells to radiation. In both *in vitro* and *in vivo* models of human cancers, including hepatocellular carcinoma[34], androgen-insensitive prostate cancer [35], oral squamous cell carcinoma [36] and lung cancer [37], some novel small molecule Aurora-A inhibitors showed radiation sensitization. However, to date, it has not been confirmed that Aurora-A overexpression results in a better response to RT in both *in vivo* and *in vitro* tumor models.

As for the reasons, we consulted a lot of related literature. Some reports have described a correlation between a better RT effect and mitotic catastrophe, which was caused by dysfunction of G2/M checkpoint regulation [38]. It is also known that Aurora-A is a key regulator of cell-cycle events from late S phase through to M phase [39], and a 2- to 6-fold increase in G2/M phase in Aurora A inhibitor-treated cells was reported compared with untreated control cells [40], whereas, the G2/M phase was most effective in radiotherapy. In addition, Aurora A inhibitors induced mitotic entry delay [41], prolonged mitotic duration [41, 42], induced mitotic spindle disassembly defects [41, 43], and cytokinesis defects [44, 45], leading to multiple centrosomes [41], and polyploid formation [23–25, 36, 41–49]. One report showed that long-term G2-arrested cells undergo senescence via G2 slippage and this cellular process of G2 slippage is the mechanism responsible for senescence of cells under long-term G2 arrest [50]. Other studies have shown that Aurora-A through a non-cell cycle-dependent method causes radiotherapy sensitization. One study [51] showed that Aurora-A enhanced the binding of NF-kappaB to DNA, thereby increasing the gene transcription by NF-kappaB and decreasing the radiosensitivity of the cells. Another study [52] showed that Aurora-A and BRCA1/2 inversely controlled the sensitivity of cancer cells to radiotherapy through the ATM/Chk2-mediated DNA repair networks.

This study demonstrated the importance of lymph node metastasis, large tumor size, and low Hb level as prognostic factors in patients with CSCC who underwent primary RT. These findings are consistent with previous reports [14, 15, 53]. However, tumor stage and treatment method failed to influence prognosis. The RTOG90-01 study [54] showed that there was no significant difference in 5-year OS and disease-free survival rates between stage III and IVA patients treated with RT compared with CCRT, similar to our results.

In the present study, we noted that the cure rate in patients with advanced CSCC who underwent definitive RT (with or without cisplatin-based chemotherapy) was not ideal as the 5-year survival rate was not high at just 27.91%. This was much lower than that reported in other studies [12, 16] at the same stage, which was approximately 50%–60%. We consider that this may have been due to the following factors: the proportion of advanced cancer patients was relatively large [stage IIB and above 92.86% (91/98) vs 46.71% (412/882)] [12], tumor volume was relatively large, and there may be a difference in Uygur patients with regard to genotype. The recurrence rate was as high as 44.96%, this percentage was much higher than that in the aforementioned studies [12, 16], which was approximately 30%. This may be due to the reasons outlined previously.

This study has several limitations. First, the relatively low number of patients and recurrences or deaths may have reduced the probability of identifying significant prognostic factors in multivariate analysis. Second, in this study, we only explored the association between survival and Aurora-A IHC staining score and did not evaluate Aurora-A expression using other techniques. Recently, Zhang et al [26] performed RT-PCR, western blot and IHC assays to determine the gene expression of Aurora-A and showed that patients with high Aurora-A expression had poorer RFS and OS rates than patients with low Aurora-A expression; multivariate analysis showed that high Aurora-A expression was an independent prognostic factor (risk ratio: 2.88; *P*=0.005). More importantly, both their study and our study showed that the Aurora-A expression level (either by RT-PCR or IHC) was an independent prognostic factor for OS.

In conclusion, although further experiments are needed to confirm these phenomena, these findings suggest that unfavorable responses to RT can be predicted based on Aurora-A overexpression in tumor cells of CSCC patients. Aurora-A overexpression was a significant prognostic factor for CSCC recurrence and was shown to be correlated with poor RFS and OS.

## MATERIALS AND METHODS

### Patients

Between January 2009 and December 2012, 129 Uyghur patients with CSCC in the People's Hospital of Xinjiang Uygur Autonomous Region were included in this study. All patients followed the principle of National Comprehensive Cancer Network definitive radical radiotherapy: radiotherapy (EBRT) combined with brachytherapy (ICRT), the prescription dose of EBRT (6 MV X-rays) was approximately 45 Gy (40–50 Gy), the prescription dose of ICRT (252Cf neutron) was approximately 40 Gy (30–40 Gy), for a total point A dose of approximately 80 Gy (70–85 Gy). Only Uyghur patients with histologically confirmed CSCC were eligible. Initially, 174 Uyghur women met the inclusion criteria, but 41 patients were excluded due to insufficient paraffin-embedded tissue and 4 were excluded due to death from a non-tumor disorder. The median age was 51.0 years (range, 33–73 years). The general physical status score was assessed according to the Eastern Cooperative Oncology Group performance status (ECOG). Classification of disease stage according to the International Federation of Gynecology and Obstetrics (FIGO) (2009) was as follows: 13 patients had stage IIA1, 5 patients had IIA2, 43 patients had IIB, 4 patients had IIIA, and 64 patients had IIIB. 74 patients were treated with CCRT (weekly cisplatin).

### Ethics and informed consent

The study protocol for the collection of tumor samples and clinical information was approved by the institutional review board, and patients provided written informed consent authorizing the collection and use of their tumor samples for research purposes. This retrospective study was carried out according to the principles set out in the Declaration of Helsinki 1964 and all subsequent revisions and was approved by the Institutional Review Board of our hospital.

### Follow up

All patients had follow-up records for over 3 years. After completion of therapy, patients were observed at 3-month intervals during the first 3 years and at 6-month intervals thereafter. OS was defined as the time from diagnosis to the date of death or when censored at the latest date if patients were still alive. RFS was defined as the length of time after diagnosis without signs or symptoms of CSCC or death. The median follow-up period was 47.0 months (range, 3–81 months). Tumor size was measured in at least five target lesions; the sum of the largest dimension was used as an initial size measurement as well as an indicator of response, as recommended by Response Evaluation Criteria In Solid Tumors criteria (RECIST) [55]. CR was defined as the complete disappearance of all measurable lesions for one month after completion of treatment. PR was defined as more than a 30% reduction in measurable lesions. PD was defined as more than a 20% increase in measurable lesions or the appearance of one or more new lesions. SD was defined as neither sufficient lesion shrinkage for PR, nor a sufficient increase for PD. Pelvic and para-aortic lymph node enlargement was defined as enlargement over a short-axis diameter of 1 cm assessed by pretreatment computed tomography or magnetic resonance imaging.

### IHC analysis and evaluation

A total of 129 formalin-fixed and paraffin-embedded tumor blocks from biopsies (collected before treatment) were obtained from the Department of Pathology, in our Hospital. To determine the expression of Aurora-A, a 4-μm section of each tumor specimen was subjected to IHC analysis. The slides were deparaffinized, rehydrated, and treated with 3% H_2_O_2_ in methanol for 15 min to inhibit endogenous peroxidase. Pretreatment was carried out in a pressure-cooker with Tris/EDTA buffer solution (pH 9.0). Following transfer to a humidified chamber, the slides were blocked with 10% normal goat serum at room temperature for 30 min and incubated with rabbit anti-Aurora-A polyclonal antibody, 1:500 (ab1287, Abcam®, Cambridge, UK) overnight at 4°C (the positive control sample was a colonic mucosal section known to express Aurora-A). In the negative controls, primary antibodies were omitted and were then incubated for 30 min at 37°C with a ready to use two-step assay kit (PV-6001, ZSGB-Bio®, BeiJing, China), followed by a DAB IHC detection kit (ZAI9017, ZSGB-Bio®, BeiJing, China) according to the manufacturer's instructions. Finally, the samples were counterstained with hematoxylin, dehydrated, and mounted. The specificity of staining was tested by selective substitution of the primary antibody by nonimmunogenic serum, and was confirmed by western blot.

Each section was rated according to the scale of intensity of staining score in addition to the area of staining. At least 10 high-power fields were randomly chosen, and >1,000 cells were counted in each section. Two independent pathologists, blinded to the follow-up data, evaluated IHC staining. A third pathologist arbitrated when discrepancies arose between these two pathologists. The intensity of the dye color was graded as 0 (no color), 1 (light yellow), 2 (light brown) or 3 (brown), and the area of staining was evaluated as follows: 0, no staining of cells in any of the microscopic fields; 1+, <30% of tissue stained positive; 2+, between 30% and 60% stained positive; 3+, >60% stained positive [38]. The two grades were added together and specimens were assigned to one of four levels as follows: a score of 0–1 (-), a score of 2 (+), a score of 3–4 (++), and a score more than 5 (+++). Immunostaining was considered to be low (-, +), or high (++, +++) (Supplementary Figure 1).

### Statistical and survival analysis

Receiver operating characteristic (ROC) curves were used to determine the best cutoff points for pretreatment Hb level, and tumor size for predicting disease recurrence. Statistical analysis of group differences was performed using the χ2 test or Fisher's exact test. Survival was estimated using the Kaplan–Meier method and the log-rank test was used to compare the survival curves. Univariate and multivariate (step-wise forward conditional method) Cox regression analyses were carried out to determine the prognostic significance of clinicopathological factors and AURKA expression. *P* <0.05 was regarded as statistically significant in two-sided tests. SPSS software (version 19.00, SPSS, Chicago, IL, USA) was used for all statistical analyses.
